# Antagonistic interaction of HIV-1 Vpr with Hsf-mediated cellular heat shock response and Hsp16 in fission yeast (*Schizosaccharomyces pombe*)

**DOI:** 10.1186/1742-4690-4-16

**Published:** 2007-03-07

**Authors:** Zsigmond Benko, Dong Liang, Emmanuel Agbottah, Jason Hou, Lorena Taricani, Paul G Young, Michael Bukrinsky, Richard Y Zhao

**Affiliations:** 1 Children's Memorial Research Center, Departments of Pediatrics, Microbiology-Immunology, Feinberg School of Medicine, Northwestern University, Chicago, Illinois, USA; 2 Departments of Pathology, Microbiology-Immunology, Institute of Human Virology, University of Maryland School of Medicine, Baltimore, Maryland, USA; 3 Department of Biology, Queen's University, Kingston, Ontario, Canada; 4 Department of Microbiology and Tropical Medicine, George Washington University, Washington, DC, USA

## Abstract

**Background:**

Expression of the HIV-1 *vpr *gene in human and fission yeast cells displays multiple highly conserved activities, which include induction of cell cycle G2 arrest and cell death. We have previously characterized a yeast heat shock protein 16 (Hsp16) that suppresses the Vpr activities when it is overproduced in fission yeast. Similar suppressive effects were observed when the fission yeast *hsp16 *gene was overexpressed in human cells or in the context of viral infection. In this study, we further characterized molecular actions underlying the suppressive effect of Hsp16 on the Vpr activities.

**Results:**

We show that the suppressive effect of Hsp16 on Vpr-dependent viral replication in proliferating T-lymphocytes is mediated through its C-terminal end. In addition, we show that Hsp16 inhibits viral infection in macrophages in a dose-dependent manner. Mechanistically, Hsp16 suppresses Vpr activities in a way that resembles the cellular heat shock response. In particular, Hsp16 activation is mediated by a heat shock factor (Hsf)-dependent mechanism. Interestingly, v*pr *gene expression elicits a moderate increase of endogenous Hsp16 but prevents its elevation when cells are grown under heat shock conditions that normally stimulate Hsp16 production. Similar responsive to Vpr elevation of Hsp and counteraction of this elevation by Vpr were also observed in our parallel mammalian studies. Since Hsf-mediated elevation of small Hsps occurs in all eukaryotes, this finding suggests that the anti-Vpr activity of Hsps is a conserved feature of these proteins.

**Conclusion:**

These data suggest that fission yeast could be used as a model to further delineate the potential dynamic and antagonistic interactions between HIV-1 Vpr and cellular heat shock responses involving Hsps.

## Background

Human immunodeficiency virus type 1 (HIV-1) viral protein R (Vpr), a virion-associated protein with a calculated molecular weight of 12.7 kilodalton (kD), is highly conserved among HIV, simian immunodeficiency virus (SIV) and other lentiviruses [1-3]. During the acute phase of the viral infection, Vpr is preferentially targeted by the HIV-specific CD8 T-lymphocytes [4,5]. Increasing evidence suggests that Vpr plays an important role in the viral life cycle and pathogenesis. For example, Vpr is required both *in vitro *and *in vivo *for viral pathogenesis and efficient viral infection of non-dividing host cells such as monocytes and macrophages [6,7]. Rhesus monkeys, chimpanzees and human subjects infected with Vpr-defective viruses have a slower disease progression often accompanied by reversion of the mutated *vpr *genes back to the wild type phenotype [8-12].

Vpr displays several distinct activities in host cells. These include induction of cell cycle G2 arrest [13-17] and cell killing [18]. The cell cycle G2 arrest induced by Vpr is thought to suppress human immune functions by preventing T cell clonal expansion [19] and to provide an optimized cellular environment for maximal levels of viral replication [8]. In addition, Vpr induces cell death, which may contribute to the depletion of CD4+ T-cells in HIV-infected patients [12,18]. Whether Vpr-induced G2 arrest and cell death are functionally independent of each other is currently of controversial. There are reports suggested that these two activities are separable both in fission yeast and mammalian cells [20-24]; others suggested that Vpr-induced apoptosis is cell cycle dependent [25,26]. Reasons for these discrepancies are not clear at the moment. In an earlier report, we demonstrated that overexpression of fission yeast (*Schizosaccharomyces pombe*) Hsp16 specifically suppresses Vpr activities, resembling cellular stress responses to heat shock, [27]. Here, we further show that this suppression is mediated by a heat shock factor (Hsf)-mediated mechanism. Furthermore, we have also tested the suppressive effect of Hsp16 on wild type and a F34I mutant Vpr. The wild type Vpr induces cell cycle G2 arrest and cell death, the F34IVpr mutant is incapable of inducing cell death but retains its ability to induce cell cycle G2 arrest both in fission yeast [21,27,28] and mammalian cells ([29]; our unpublished data) Thus, examination of the wild type and the F34I mutant Vpr enable us to investigate these two Vpr activities separately. In addition, the highly conserved Vpr effect on cell cycle G2/M regulation and cell survival makes fission yeast a particularly useful model to study mechanisms of these Vpr activities (For review of this subject, see [30-34]). Interestingly, v*pr *gene expression appears to trigger a moderate increase in Hsp16 levels but counteracts heat shock-mediated elevation of Hsp16. Together, our findings suggest a highly conserved and dynamic interplay between *vpr *gene expression and cellular heat shock response involving heat shock proteins.

## Results

### Endogenous Hsp16 is responsive to vpr gene expression

**Figure 1:**
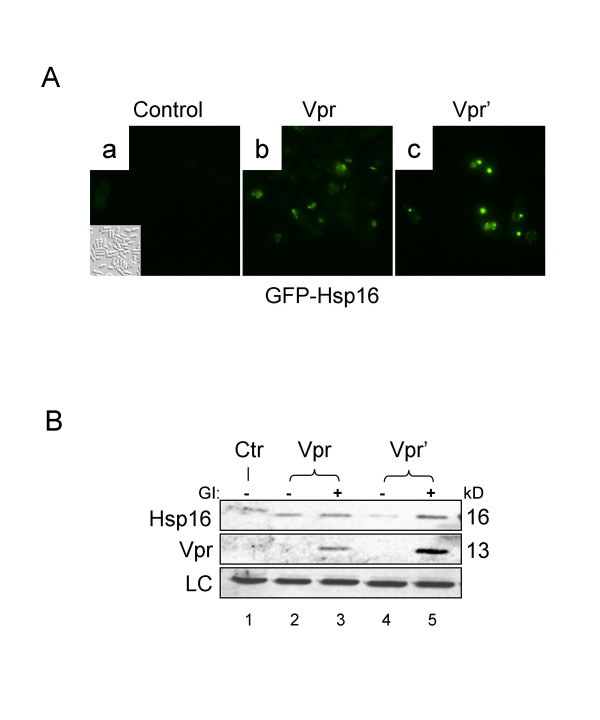
**Endogenous Hsp16 is responsive to *vpr* gene expression. **   (A) Expression of *hsp16* was measured through GFP green fluorescence as shown by gfp-hsp16 fusion protein expression.  Cells were grown under normal growth conditions and expression of the wild type *vpr* (Vpr) or mutant F34I *vpr* (Vpr’) was induced in thiamine depleted EMM medium as previously described [35].  Photographs were taken 24 hrs after gene induction.  Small panel in A-*a* shows cells without green fluorescence.  (B) Comparison of the Hsp16 protein levels in the presence and absence of Vpr as shown by Western blot analysis.  The *vpr* or *vpr’*-expressing cells were collected at the same time as in panel (A).  Lane 1 shows wild type SP223 cells without plasmid; lanes 2 and 4 show cells with vpr gene expression repressed; lanes 3 and 5 – cells with *vpr* gene expression induced.  Note that elevation of Hsp16 shown in lane 2 is most likely due to leakage of *nmt1* promoter and low level gene expression under these conditions [36].  LC, protein loading control.  A protein band that nonspecifically reacted to the antibody was used as a protein loading control. GI, gene induction.

We previously identified fission yeast Hsp16 as a potent Vpr suppressor [27]. Analysis of *hsp16 *expression in *S. pombe *Q1649 strain, in which the *hsp16 *gene is tagged with GFP and is under the control of its native promoter [35], demonstrated that both the wild type Vpr and the mutant protein (Vpr') elicited Hsp16 production (Fig. 1A). The mutant Vpr', in which phenylalanine in position 34 was replaced with isoleucine (F34IVpr), was used in this study to measure Vpr-induced cell cycle G2 induction because the wild type Vpr kills cells. Vpr' has lost its ability to induce cell killing but retains its capacity to induce G2 arrest as previously shown both in human (our unpublished data) and yeast cells [21,27,28].

We next tested whether the expression of endogenous *hsp16 *is responsive to *vpr *gene expression. Both the wild type *vpr *and F34I mutant *vpr *genes were induced by depleting thiamine from the EMM medium as previously described [36,37]. As shown in Fig. 1B, expression of wild type *vpr *or mutant *vpr' *under normal growth conditions elicited a moderate increase of the Hsp16 protein level (Fig. 1B, lanes 3 and 5). The faint protein band in lane 2 could possibly be due to low level of *vpr *expression even when the inducible promoter is repressed [36]. Together, these observations suggest that Hsp16 production is responsive to *vpr *gene expression. These results are consistent with our studies in mammalian cells where *vpr *gene expression stimulates expression of *HSP27*, a human paralogue of Hsp16 (Our unpublished data).

### Overproduction of Hsp16 suppresses viral infection in CD4-positive T-cells and macrophages

Vpr activities have been implicated as positive factors for HIV-1 replication [6,8,38]. Consistent with these activities, Vpr has been shown to increase viral replication 2 to 4 fold in proliferating T lymphocytes [8,39,40] but its activities are required for viral infection in non-dividing cells such as macrophages [6,7]. Responsive expression of human *HSP27 *and yeast *hsp16 *to Vpr suggest a possible and highly conserved cellular activity against Vpr. Indeed, we have showed previously that overproduction of Hsp16 reduces viral replication in CD4-positive T-cells in a Vpr-dependent manner [27].

**Figure 2:**
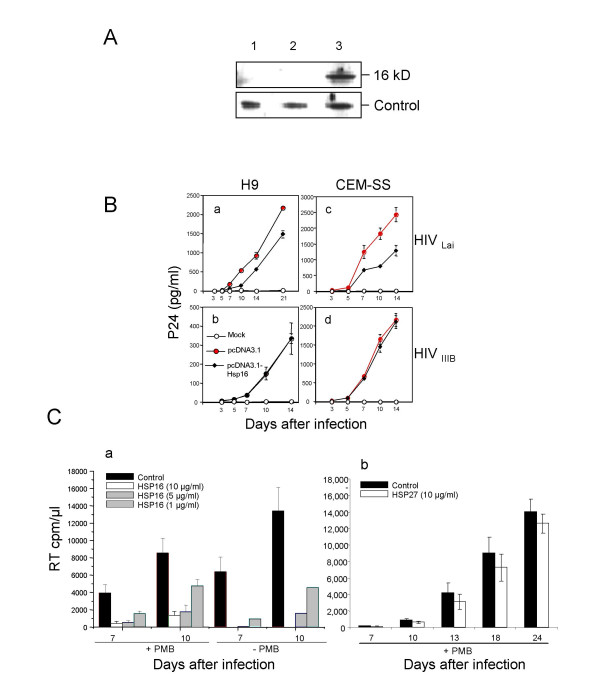
**Hsp16 suppresses HIV-1 replication in CD4+ T-lymphocytes and macrophages**. (A, B) Effect of Hsp16 on HIV-1 replication in CD4+ T-lymphocytes.  (A) Western blot analysis shows level of Hsp16 in HIV-infected CD4+ H9 cells.  Lane 1, mock-infected H9 cells; lane 2, HIV-infected H9 cells carrying empty vector pcDNA3.1; lane 3, HIV-infected cells *hsp16*-expressing plasmid.  Control, protein loading control.  (B) Suppression of HIV-1 viral replication by Hsp16 requires C-terminal end of Vpr.  3 x 10^6^ to 5 x 10^6^ of *hsp16*-expressing H9 or CEM-SS cells were either mock infected or infected with 2.0 x 10^3^ TCID50 of HIV-1LAI or IIIB.  Equal infection of the cells was further verified by measuring viral RNA levels 24 hr after viral inoculation.  Viral replication was determined by p24 antigen levels.  (C) Analysis of Hsp16 or HSP27 effects on HIV-1 replication in macrophages.  Increasing concentration (1, 5 or 10 μg/ml) of purified recombinant fission yeast Hsp16 or 100 μg/ml of recombinant human HSP27 protein was added to 1 x 10^6^ primary human macrophages infected with HIV-1ADA.. Viral inoculates were equalized according to reverse transcriptase (RT) activity (5 x 10^5^ counts per minute/10^6^ cells.  Viral replication was monitored 7 and 10 days after infection by measuring reverse transcriptase (RT) activity in culture supernatants.  To neutralize the effect of potential contamination with endotoxin [45], 10 μg/ml of Polymyxin B (PMB) was added [45].  Results are mean ± SE of triplicates.

To further delineate the suppressive effect of Hsp16 on Vpr, here we tested the effect of Hsp16 on viral replication in CD4-postive cells infected by a viral strain IIIB, in which the *vpr *gene has a frame shift mutation at codon 73 resulting in a truncated Vpr protein that misses 24 a.a. at its C-terminus [8,11,41]. The C-terminal Vpr is responsible for a number of Vpr activities including protein dimerization [42], cell cycle G2 arrest and cell death [20,43]. We established a CD4+ H9 cell line stably producing high level of yeast Hsp16 (Fig. 2A). These H9 cells were then infected with a HIV-1 Vpr-positive laboratory strain LAI. To test the potential effect of Hsp16 on viral replication, p24 antigen was measured in culture supernatants over a period of 21 days after infection. As shown in Fig. 2B and consistent with our previous findings [27], a consistent but moderate reduction of HIV-1 viral replication was observed in cells expressing *hsp16*. For example, levels of p24 antigen steadily increased in HIV-infected cells expressing the vector control from day 3 to day 21 of HIV-1 infection indicating successful viral infection. (Fig. 2B-a). However, a 1.5 to 4.5-fold reduction in p24 antigen levels was detected in HIV-infected cells expressing Hsp16 from day 10 to 21 after viral infection. No detectable p24 antigen was observed in mock-infected cell over the entire experimental period. To ensure the observed viral inhibition by Hsp16 is not cell line-specific, we examined another CD4-positive cell line, CEM-SS, which was also derived from T lymphocytes [44]. A similar suppressive effect on viral replication (1.5 to 3.1-fold reduction) was also observed in the CEM-SS cells that stably express *hsp16 *genes (Fig. 2B–c).

To examine whether Hsp16 retains its suppressive effect on viral replication when 24 aa of the C-terminal Vpr is removed, we repeated the same infection experiments in the H9 and CEM-SS cells using the C-terminal truncated Vpr-carrying viral strain IIIB. As shown in Fig. 2B-b and Fig. 2B–d, the kinetics of viral replication were essentially indistinguishable between cells with or without Hsp16, suggesting that Hsp16 has lost its inhibitory effect on viral replication in the absence of C-terminal end of Vpr.

The above data suggest the suppressive effect of Hsp16 is specific to Vpr. Since Vpr is required for viral infection in non-dividing cells such as macrophages, we next tested the potential effect of Hsp16 on HIV infection in macrophages. Purified fission yeast Hsp16 protein was added to primary human macrophages infected with HIV-1_ADA _with increasing concentration from 1, 5 to 10 μg/ml of cells. Viral replication was followed 7 and 10 days after infection by measuring the reverse transcriptase (RT) activities in culture supernatants. To avoid potential interference of endotoxin that often presents in purified recombinant proteins [45], purified Hsp16 was treated with 10 μg/ml Polymyxin B (PMB)-agarose that was shown to efficiently remove endotoxin [45]. As shown in Fig. 2C-a, infected macrophages without removing endotoxin (-PMB) almost completely eliminated viral replication at day 7 after infection; about 3.5 to 7-fold decrease of viral infection was observed at day 10 with 1 or 5 μg/ml of Hsp16. No viral activity was detectable at 10 μg/ml level. After removing the possible endotoxin from Hsp16, reduced but still significant reduction of viral replication was observed both at day 7 and day 10 after infection. 2.6 to 8.0-fold decrease of viral replication were seen at day 7 with 1.7 to 5.5-fold reduction of viral replication was observed in day 10. These data suggest a dose-dependent suppression of viral replication by Hsp16 in macrophage. To ensure the observed effect was indeed due to Hsp16, as a control, we also tested the potential effect of purified HSP27. 10 μg/ml of HSP27 with the same level of PMB (10 μg/ml) was added to HIV-1_ADA_-infected macrophages the same way as we did for Hsp16. RT activities were measured over time. As shown in Fig. 2C-b, no significant differences were seen during the entire 24 days after infection. These data suggest a dose-dependent suppression of viral replication by Hsp16 in macrophage. Together, these data show that overproduction of Hsp16 specifically inhibits HIV-1 infection possibly by targeting the Vpr activates.

### Heat shock factor is the key regulator for the elevation of Hsp16 and heat shock-mediated suppression of Vpr

**Figure 3:**
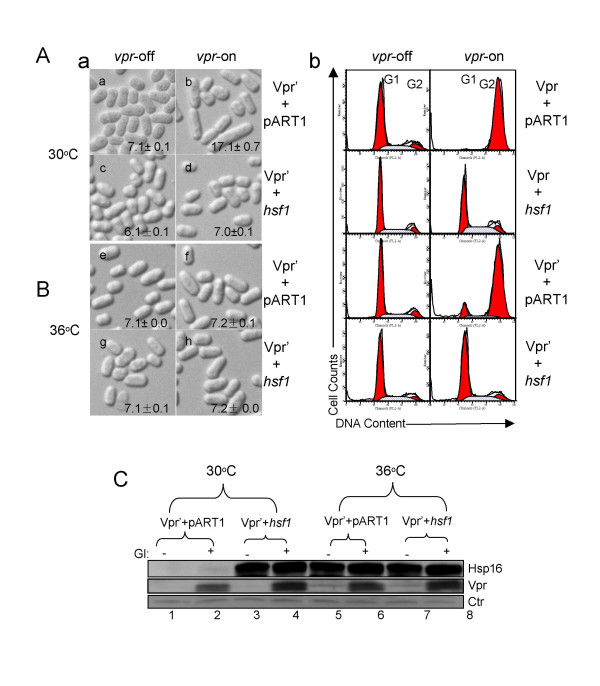
**Heat shock factor is responsible for Hsp16 elevation and heat shock-mediated suppression of the Vpr activities**. (A) Overexpression of hsf1 suppressed Vpr-induced G2 arrest. *a*, overexpression of *hsf1* reduced Vpr’-induced cell elongation back to normal size. Cell images were captured 30 hrs after gene induction.  *b*, overexpression of *vpr* or *vpr’* suppressed Vpr-induced G2 arrest as measured by flow cytometric analysis.  The *vpr*-repressing (off) and *vpr*-expressing (on) cells were prepared as described previously [37].  Forty-eight hours after vpr gene induction, cells were collected for flow cytometric analysis.  (B) No additional reduction of Vpr-induced cell elongation was seen when *hsf1*-expressing cells were treated with high temperature (36°C).  Average and standard deviation of cell length was calculated based on three independent experiments by counting a minimum 90 cells.  (C) Inducible expression of HIV-1 *vpr’* and constitutive expression of *hsf1* under normal (30°C) and high (36°C) temperature shown by Western blot analysis.  Proteins were extracted from cells cultured 30 hrs after gene induction (GI).  Because of the lack of an antibody against fission yeast anti-Hsf1 and our interest in monitoring Hsf1-mediated Hsp16 elevation, Hsp16 protein production was used here as marker for Hsf1 activity [35].

Overexpression of *hsp16 *by itself has no any obvious effect on cell length or morphology [27,35]. However, our earlier data showed that overexpression of *hsp16 *or high temperature (36°C) suppressed Vpr-induced G2 arrest as measured by cell elongation in fission yeast [27], indicating a potential and specific suppressive effect of Hsp16 on Vpr. Since Hsp16 can be activated by host cellular stress responses through heat shock factor (Hsf)-mediated pathway, we next investigated the potential involvement of heat shock factor (Hsf) in the heat shock-mediated suppression of Vpr. There is only one Hsf in *S. pombe*. However, deletion of *hsf1 *is lethal in yeast [46], thus we were unable to test the deletion effect of Hsf on the Vpr activities. Instead, we overexpressed the *hsf1 *gene from a pART1-*hsf1 *plasmid where it is controlled by an exogenous and constitutively expressing *adh *promoter. Since no specific antibody against Hsf1 is available, we used Hsp16 as a marker for *hsf1 *expression [35]. As shown in Fig. 3A-*a*, *b*, empty pART1 plasmid had no effect on Vpr'-induced cell elongation. Cells were 17.7 ± 0.7 μm in length 30 hrs after *vpr *gene induction [47]. Expression of *hsf1 *by itself in *S. pombe *cells gave rise to slightly shorter (6.1 ± 0.1 μm) than normal cells (7.1 ± 0.1 μm) (Fig. 3A-*c*). Significantly, expression of *hsf1 *appeared to prevent Vpr'-induced cell elongation. Cell length measurements for *hsf1 *and *vpr'*-expressing cells had an average of 7.0 ± 0.1 μm, which is indistinguishable from the normal cells without Vpr' and elevated Hsf1 (7.1 ± 0.1 μm; Fig. 3A-*d*). Western blot analysis confirmed proper Vpr' protein production under the inducible condition and the Hsf-mediated production of Hsp16 in these cells (Fig. 3C, lane 3–4). Western blot analysis further verified that *vpr *gene expression was not affected by Hsf1 expression (Fig. 3C, lane 4 *vs*. lane 2).

These observations suggested that Hsf1 is probably the major cellular factor that contributes to the anti-Vpr activities. To verify this finding, we further examined whether heat shock treatment can induce additional shortening of cells besides the suppressive Hsf1 effect. If additional cellular factors are involved in suppressing Vpr' during the cellular heat shock response, we would expect to see shorter cell length than when Hsf is overexpressed alone. The same experiment as described above was repeated at elevated temperature (36°C). No additional shortening of cells beyond the length observed with Hsf1 overexpression at normal temperature was seen when *vpr*-expressing cells were grown at 36°C with overproduced Hsf1 (Fig. 3B). Together, results of these experiments suggest that Hsf is the key cellular regulator of heat shock-mediated suppression of the Vpr activities.

### Vpr counteracts Hsp16 elevation induced by heat treatment at the transcriptional level

**Figure 4:**
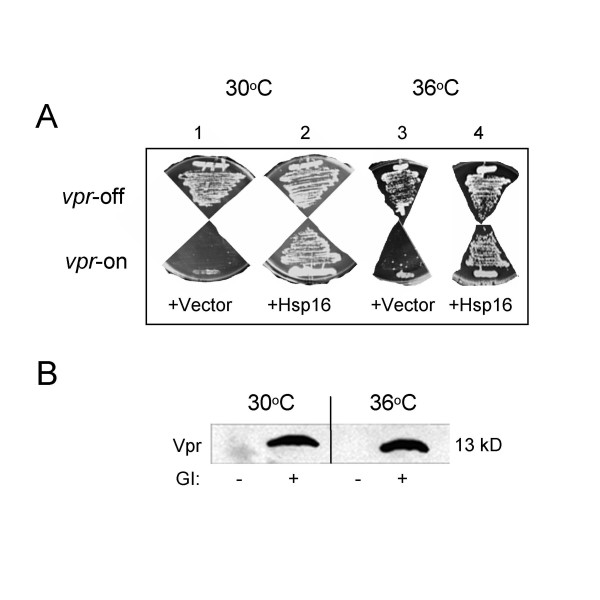
**Expression of *hsp16 *under exogenous *nmt1 *promoter rather than endogenous promoter suppresses Vpr-induced cell killing**. (A) High temperature does not suppress Vpr-induced cell killing but expression of *hsp16* through a foreign *nmt1* promoter does.  Plates in the top row are fission yeast cells streaked on thiamine-containing (*vpr*-off) EMM plates; bottom row plates are the same as the top plates except no thiamine (*vpr*-on) was added.  Plates are shown after 3-5 days of incubation.  (B) Induction of cellular heat shock responses does not affect the protein levels of the wild type Vpr (RE007) as indicated by the Western blot analysis.  GI, gene induction.

**Figure 5:**
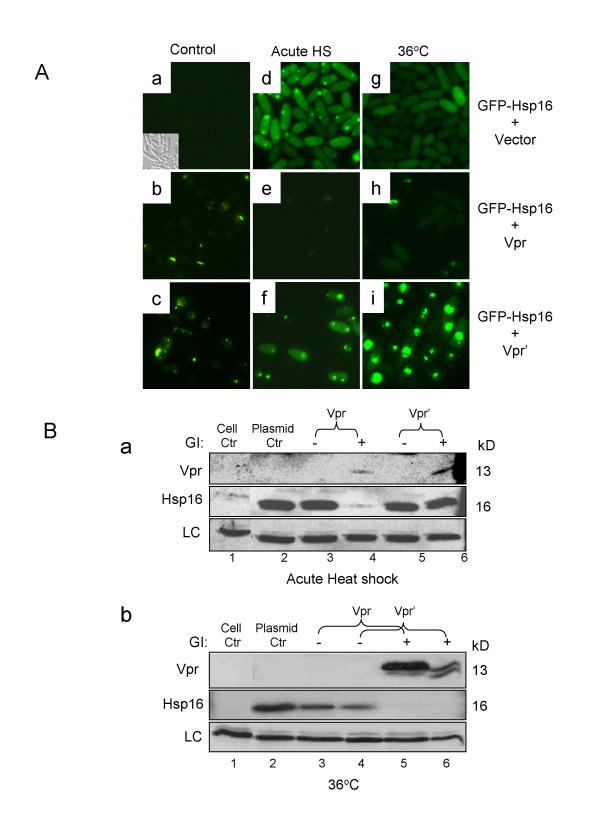
**Down-regulation of Hsp16 activation by Vpr**. (A) Expression of *hsp16* monitored by GFP-Hsp16 fusion protein expression.  For heat shock treatment, *vpr* gene expression was first induced at 30ºC for 21 hours and then cultures were treated with acute heat shock (Acute HS) at 45ºC for 15 min (middle columns) or exposed to constant 36°C (right columns).  The level of Hsp16 expression was examined 2 hrs after the heat shock, i.e., 23 hrs after *vpr* gene induction (GI).  (B) Comparison of the Hsp16 protein levels between acute heat shock and constant heat treatment shown by Western blot analysis.  The *hsp16*-expressing *vpr* (RE007) and vpr’ (RE076) cells were collected at the same time as in (A), i.e., 23 hrs after vpr gene induction.  *a*, Hsp16 protein levels under acute heat shock conditions.  Acute heat shock (45ºC for 15 min) was used to transiently activate Hsp16, and the Vpr effect was measured 2 hrs after the heat shock.  *b*, Hsp16 protein levels under constant and prolonged high temperature at 36°C.  The effect of Vpr on Hsp16 was measured 48 hrs after cell culturing at 36ºC, which normally induces constant elevation of Hsp16.  Lane 1 shows wild type SP223 cells without plasmid (Cell Ctr); lane 2 shows SP223 cells carrying an empty plasmid (Plasmid Ctr); Ctr, control; GI, gene induction, +, *vpr*-on; -, *vpr*-off.  LC, a protein band that non-specifically reacted to the antibody and was used as a protein loading control.

Even though induction of cellular heat shock response by heat treatment suppresses *vpr'*-induced cell cycle G2 delay, surprisingly, the same heat treatment was not able to block Vpr-induced cell death in RE007 cells which express the wild type *vpr *(Fig. 4A-3, bottom plate). Inability of colony formation at high temperature is not due to lack of *vpr *expression because Western blot analysis showed that heat treatment does not affect the Vpr protein level (Fig. 4B; [27]). Since heat treatment induces high levels of Hsp16 [35] and artificial overproduction of Hsp16 suppresses Vpr-induced cell death at both temperatures (Fig. 4A-a and 4A-4, bottom), it was puzzling why heat treatment only suppresses Vpr-induced G2 arrest but it does not suppress Vpr-induced cell killing. One potential explanation is that wild type Vpr may actually prevent heat-induced elevation of Hsp16. To test this possibility, we measured protein levels of Hsp16 in the presence and absence of Vpr using different methods. One was to observe the fluorescent signal emitted by the GFP-Hsp16 fusion protein in a *S. pombe *Q1649 strain, in which the *hsp16 *gene is tagged with GFP and is under the control of its native promoter (Fig. 5A; [35]). Changes in the Hsp16 protein level were further quantified by measuring fluorescent intensity (FI) using a luminescence spectrophotometer [35,48,49]. In addition, Western blot analysis was also carried out to measure endogenous Hsp16. Two heat treatment methods were used to delineate the potential effect of Vpr on the Hsp16 protein levels. Acute heat shock (45°C for 15 min) was used to transiently activate Hsp16, and the Vpr effect was measured 2 hrs after the heat shock. As an alternative method, constant high temperature was used for lasting elevation of Hsp16, and the effect of Vpr on Hsp16 was measured 48 hrs after cell culturing at 36°C.

Under the normal growth conditions, Hsp16 protein expression is typically very low or undetectable (FI = 0.1 ± 0.3; Fig. 5A-a; Fig. 5B-a,b, lane 1 [35]). When these cells were subjected to an acute heat shock (45°C for 15 min), a significant increase (FI = 5.9 ± 0.2) in the Hsp16 protein level was observed 2 hr after heat shock in cells that either had no *vpr*-containing plasmid (Fig. 5A-*d*; Fig. 5B-*a*, *b*, lane 2) or *vpr *gene expression was suppressed (Fig. 5B-*a*, lanes 3,5). In contrast, the level of Hsp16 (FI = 3.1 ± 0.6) was markedly decreased when wild type *vpr *was expressed under the same heat shock conditions (Fig. 5A-*e*; Fig. 5B-*a*, lane 4). Similar Hsp16 elevation (Fig. 5A-*g*; Fig. 5B-*b*, lane 2–4) was also observed in cells grown under constant high temperature at 36°C. Consistent with the observation shown in acute heat shock experiment, Hsp16 protein level was diminished in the *vpr*-expressing cells cultured at 36°C for 48 hrs (Fig. 5B-b, lane 5). Thus, wild type Vpr indeed inhibited heat-mediated activation of Hsp16. Interestingly, no obvious decrease of Hsp16 was observed with the RE076 cells carrying a mutant Vpr' during the early hours (23 hrs) of heat treatments (Fig. 5A-*i*; Fig. 5B-*a*, lane 6).

However, after prolonged (48 hrs) incubation of *vpr*-expressing cells at constant high temperature, both the wild type and mutant Vpr were able to eliminate Hsp16 elevation (Fig. 5B-*b*, lane 5–6). Taken together, these observations provide an explanation to our finding that heat treatment suppresses the Vpr'-induced cell cycle defect but does not protect against Vpr-induced cell killing because the F34I mutation in Vpr' may have attenuated the ability of Vpr to down-regulate Hsp16 thus allowing elevated Hsp16 to suppress activity of Vpr'. Therefore, wild type Vpr specifically counteracts activation of Hsp16 in response to *vpr *gene expression or heat treatment.

It is of interest to note that overexpression of *hsp16 *under the control of an exogenous *nmt1 *promoter suppressed Vpr-induced cell killing in the wild type cells (Fig. 4A-2 bottom panel; [27]) suggesting that the counteracting effect of Vpr on Hsp16 is specifically targeted to the *hsp16 *promoter, i.e., occurs at the transcriptional level. Attempting to confirm this possibility, we further tested whether overexpression of *hsp16 *under the same *nmt1 *promoter was also capable of suppressing Vpr-induced cell death at 36°C when Vpr has the strongest counteracting effect on Hsp16. As shown in the bottom panel of Fig. 4A-4, overproduction of Hsp16 was indeed capable of blocking Vpr-induced cell death at both high (36°C) and normal growth temperature (30°C). Therefore, Vpr counteracts Hsp16 elevation induced by heat treatment most likely at the transcriptional level.

## Discussion

In this report, we provide evidence that Hsf1 is the main regulator responsible for Hsp16 elevation and anti-Vpr responses in fission yeast cells. The fact that Hsf1 is responsible for Hsp16-mediated response to Vpr indicates that the effect of Hsp16 on Vpr resembles the cellular heat shock responses. Indeed, overexpression of *hsf1 *completely reduced Vpr'-induced cell cycle G2 arrest as shown by reversion of the cell elongation (Fig. 3A-*d *vs. *b*), shift of the cellular DNA content from G2 to G1 (Fig. 3A-*b*) and additional heat treatment of *hsf*-expressing cells did not significantly enhance the suppressive effect of Hsf1 on Vpr (Fig. 3B-*h *vs. *d*).

Even though *vpr *gene expression triggers Hsp16 elevation, Vpr appears to prevent further elevation induced by heat treatment (Fig. 5A-*e*; Fig. 5B-*a*, lane 4; Fig. 5B-*b*, lane 5), suggesting a counteracting effect of Vpr on heat stress-like cellular response possibly through transcriptional regulation of *hsp16*. This notion is supported by our observations that induction of Hsp16 by heat treatment failed to counteract Vpr-induced cell death (Fig. 4A-3, bottom plate). However, overexpression of *hsp16 *under the control of an exogenous *nmt1 *promoter completely suppressed Vpr-induced cell death under the same heat shock conditions ([27]; Fig. 4A-4). Possible transcriptional down-regulation of *hsp16 *by Vpr is further evidenced by the results shown in Fig. 5A, in which a GFP reporter was fused with the endogenous *hsp16 *promoter [35] and expression of *vpr *eliminated the Hsp16 elevation (Fig. 5A-*e*, *h*). Although the molecular mechanism underlying this transcriptional suppression of *hsp16 *is unclear at the moment, the fact that Hsf activates Hsps through binding of the Hsp promoters [50] and overexpression of *hsf1 *or *hsp16 *through an exogenous *adh *or *nmt1 *promoter alleviates the Vpr activity (Fig. 3A; [27]) support the idea that Vpr may affect expression of *hsp16 *through competition with Hsf for control of *hsp16 *expression. One possible scenario is that Vpr may inhibit Hsf1 that results in reduced transcription of *hsp16*. Alternatively, since Vpr is a weak transcriptional activator through binding to the transcriptional factor Sp1 [51], it is also possible that Vpr may compete with Hsf1 by binding to the Sp1 region of the *hsp16 *promoter. Obviously additional tests are needed to elucidate these possibilities. Interestingly, only the wild type Vpr was able to inhibit Hsp16 at early hours (23 hrs) after induction, as a single amino acid substitution from phenylalanine to isoleucine at position 34 of Vpr attenuated its ability to suppress the increase of Hsp16 after acute heat shock (Fig. 5A-*i *vs. *h*; Fig. 5B, lane 6 *vs. *lane 4). In fact, an even higher level of Hsp16 was observed. This is presumably due to the inability of Vpr' to compete with Hsf-mediated Hsp16 elevation. Thus, assuming that expression of *hsp16 *is responsive to both the presence of Vpr and heat shock treatment, this larger increase of Hsp16 could be an additive effect. Since amino acid substitution at residue 34 of Vpr diminishes the ability of Vpr to induce cell death but retains induction of G2 arrest [20,28,29], a plausible possibility is that suppression of Hsp16 and induction of cell death by Vpr share common pathways.

It should be mentioned that whether Vpr-induced G2 arrest and cell death are two functionally independent activities is still of debate. Earlier reports suggested that these two activities are separable both in fission yeast and mammalian cells [20-24][52]. However, recent reports indicated Vpr-induced apoptosis is cell cycle dependent [25,26]. Although reasons for these discrepancies are not completely clear at the moment, it is noticed that apoptosis shown in the Andersen's study describes a late event as cells were collected 48–72 hrs after viral infection [26]. Prolonged cell cycle G2 arrest results in apoptosis. Thus, it is not surprising to find that apoptosis described in the Andersen's study is ANT-independent and ANT-dependent apoptosis was documented previously [53]. Additional difference between the apoptosis described by Andersen et al from others is also noticed in the examination of two Vpr mutations. The R77Q and I74A mutants, which separate the apoptosis and G2 arrest induced by Vpr [24,54], showed no separation between the G2 induction and apoptosis. In our study, the F34IVpr mutant is unable to induce cell death but retains its ability to induce cell cycle G2 arrest both in fission yeast [21,27,28] and mammalian cells ([29]; our unpublished data) It thus allowed us to differentiate the effect of a wild type Vpr vs. a mutant Vpr that only confers the inhibitory effect on cell cycle regulation.

Responsive elevation of fission yeast Hsp16 and its human paralogue HSP27 (our unpublished data) suggests that the cellular heat stress-like responses might be antagonistic to Vpr. Indeed, we previously showed that overexpression of *hsp16 *and human *HSP27 *suppress the Vpr activities, including cell cycle G2 arrest and cell killing, both in fission yeast and human cells ([27]; our unpublished data). However, the suppressive effect of yeast Hsp16 and human HSP27 on Vpr are not identical. Overproduction of Hsp16 completely eliminated all of the Vpr activities including the positive role of Vpr in supporting viral replication in macrophages (Fig. 2C-*a*). Under the same condition, however, HSP27 has no clear suppressing effect against Vpr in macrophages (Fig. 2C-*b*). One possible difference between these two HSPs is that Hsp16 associates directly with Vpr [27] but no clear HSP27-Vpr interaction was detected both *in vitro *and *in vivo *(our unpublished data). Unlike Hsp16, overexpression of *HSP27 *is unable to block nuclear transport capacity of Vpr (our unpublished data). Since nuclear transport of Vpr is required for HIV-1 infection in non-dividing cells such as macrophages [7,55,56], the inability of HSP27 to block nuclear import of Vpr could potentially explain why it has no effect on HIV-1 infection in macrophages.

There appears to be a dynamic interaction between *vpr *gene expression and activation of Hsp16 in fission yeast. Results of our parallel studies in mammalian cells indicated a similar dynamic and antagonistic interaction between Vpr and HSP27 (our unpublished data). This finding is not surprising because activation of heat shock proteins by Hsf1 is a highly conserved cellular process among all eukaryotic cells [50]. All eukaryotes encode at least one heat shock factor that is believed to regulate transcription of heat shock genes. This protein binds to a regulatory sequence, i.e., the heat shock element, that is absolutely conserved among eukaryotes [50]. Based on the data presented, we hypothesize that expression of *vpr *or HIV infection elicits a transient activation of the small heat shock proteins (sHsps) of eukaryotes through an Hsf-mediated pathway. Activation of these sHsps is most likely a part of the cellular antiviral reaction to HIV infection and specifically to Vpr. However, these stress responses are normally not sufficient to suppress the Vpr activities because of active counteraction from Vpr. Importantly, however, the Vpr activities could be completely blocked when sHsp's are produced under control of an exogenous promoter thus avoiding transcriptional inhibition by Vpr. Since the Vpr-specific activities have been linked to such clinical manifestation of AIDS as activation of viral replication [57], suppression of host immune responses [19] and depletion of CD4+ T-lymphocytes [12,58], this finding could potentially provide a new approach to reducing Vpr-mediated detrimental effects in HIV-infected patients by stimulating expression of sHsps.

## Methods

### Maintenance and growth of mammalian and yeast cells

Genotypes and sources of *S. pombe *strains, mammalian cell lines and plasmids used in this study are summarized in Table 1. CD4-positive H9 and CEM-SS cells were grown in RPMI 1640 medium supplemented with 10% heat-inactivated fetal calf serum (FCS) and 100 unit/ml of penicillin/streptomycin. Gene inductions of HIV-1 *vpr *and other cellular genes under the control of the *nmt1 *promoter in fission yeast have been described previously [27,37]. Cells containing the plasmid with the *nmt1 *promoter were first grown to stationary phase in the presence of 20 μM thiamine. Cells were then washed three times with distilled water, diluted to a final concentration of approximately 2 × 10^5 ^cells/ml in 10 ml of the appropriately supplemented EMM medium with or without thiamine. Cells were examined approximately 24 hours after gene induction. Fission yeast cells were normally grown at 30°C with constant shaking at 250 rpm unless otherwise specified.

Induction of cellular heat shock responses were conducted as previously described [27,35,59]. Briefly, cultures were first grown as mentioned above to fully express *vpr *and then exposed to either an acute heat shock at 45°C for 15 min or grown at consistent high temperature at 36°C for an indicated period of time.

### Measurement of the Vpr-specific activities in fission yeast

All of the functional assays used to measure Vpr-specific activities, i.e. cell cycle G2 arrest and cell death induced by Vpr, have been described previously [20,37,60]. Vpr-induced G2 arrest can be specifically measured in fission yeast based on a number of cellular endpoints [21,37]. Here we used F34IVpr (Vpr')-induced cell elongation as a marker of G2 arrest [47,61]. The mutant Vpr', in which phenylalanine was replaced with isoleucine in position 34 (F34IVpr) was used instead of the wild type Vpr because Vpr' has lost its ability to induce cell killing but retains its capacity to induce G2 arrest, as previously shown both in human (our unpublished data) and fission yeast cells [21,27,28]. The use of cell elongation, also known as "cdc phenotype", as a marker for cell cycle G2/M delay is a standard approach in fission yeast [47,61-63]. Cell images were first captured on a Leica microscope and the cell length was determined using OpenLab software. Average and standard deviation of cell length were calculated based on three independent experiments, each counting at least 100 cells. To verify Vpr-induced cell cycle G2 arrest, flow cytometric analysis is carried out as previously described [37]

Induction of cell death by Vpr was detected by inability of *vpr*-expressing cells to form colonies on agar plates as previously described [60]. Briefly, *S. pombe *cells containing the pYZ1N::*vpr *constructs were first grown on a selective leucine-free minimal EMM plate under *vpr*-repressing condition. A loopful of viable cells was streaked onto *vpr*-inducing or *vpr*-repressing EMM plates and incubated at 30°C for 3–4 days. Inability to form colonies on the *vpr*-inducing EMM plates but normal growth on the *vpr*-repressing EMM plates is indicative of Vpr-induced cell killing [20,64].

### Viral infections

To evaluate the suppressive effect of Hsp16 on viral replication in proliferating CD4-positive T-lymphocytes, H9 and CEM-SS cells [41] that stably express a plasmid control or *hsp16 *were established [27]. 3 × 10^6 ^to 5 × 10^6 ^of these H9 or CEM-SS cells were either mock infected or infected with 2.0 × 10^3 ^TCID_50 _of HIV-1_LAI _or IIIB. The HIV-1 LAI strain carries a wild type *vpr *gene; the *vpr *gene in the IIIB strain has a frame shift mutation at codon 73, which results in a truncated Vpr protein missing its C-terminus [8,11,41]. Equal infection of the cells was further verified by measuring viral RNA levels 24 hr after viral inoculation using the Roche Monitor assay following the manufacturer's instructions. Viral replication was determined by p24 antigen levels using a commercially available HIVAG-1 polyclonal antigen kit (Abbott Laboratories, Abbott Park, IL).

Monocyte-derived macrophages (MDMs) were prepared from peripheral blood mononuclear cells (PBMCs) by adherence to plastic as described previously [65]. Briefly, human PBMCs were isolated from buffy coats of healthy seronegative donors by Ficoll density gradient centrifugation (Ficoll-Paque PLUS; Pharmacia Biotech, Piscataway, NJ). PBMCs were cultured in Primaria flasks (Becton Dickinson, Franklin Lakes, NJ) in culture medium (Dulbecco modified Eagle medium [DMEM]) supplemented with 10% heat-inactivated normal human serum, 2 mM glutamine, 50 U/mL penicillin, and 50 μg/mL streptomycin (all from Life Technologies, Bethesda, MD) at 37°C in a CO_2 _incubator at a cell density of 8 × 10^6 ^cells/mL. At 2 hours after plating, nonadherent cells were aspirated, and the adherent cells were cultured overnight in DMEM supplemented with macrophage colony-stimulating factor (M-CSF, 2 ng/mL). At 18 hours after initial plating, adherent cells were detached with 10 mM EDTA (ethylenediaminetetraacetic acid)/phosphate-buffered saline (PBS) and plated in 24-well Primaria plates (Becton Dickinson) at a density of 10^6 ^cells/mL for 7 days in the presence of recombinant human M-CSF (at 2 ng/mL). After 7 days, final MDM cultures were composed of about 98% macrophages as judged by morphology and nonspecific esterase activity. A macrophage-tropic HIV-1 strain ADA [66] was used for infection of MDM. Cells were infected with viral inoculae equalized according to the reverse transcriptase (RT) activity (5 × 10^5 ^counts per minute/10^6 ^cells).

### Fluorescence microscopy

A Leica fluorescence microscope DMR equipped with a high performance CCD camera (Hamamatsu) and OpenLab software (Improvision, Inc., Lesington, MA) was used for all imaging analyses. Fission yeast cells were collected onto a regular glass slide and covered with cover slip. For the observation of green fluorescent protein, we used a Leica L5 filter, which has an excitation of 480/40 (460–500 nm) and emission of 527/30 (512–542 nm). Induction of cellular heat shock responses were conducted as previously described [35,59]. The level of *hsp16 *gene expression was quantified by measuring fluorescent signal emitted from the GFP-Hsp16 fusion protein as described previously [35,48,49]. For DNA staining, cells were counterstained with 1 μg/ml DAPI, which was observed with a Leica A8 filter with an excitation of 360/40 (340–380 nm) and emission of 470/40 (450–490 nm). Cell length was measured individually on the captured images using the OpenLab software. Statistical significance of differences in cell length was determined using the *t*-test for paired samples.

### Western blot analysis

For Western blot analysis, mammalian cells were harvested and rinsed with ice-cold HEPES-buffered saline (pH 7.0), then lysed in an ice-cold cell lysis buffer [20 mM Tris-HCl, pH7.6, 150 mM NaCl, 1 mM EDTA, 0.5% Nonidet P-40, 1 mM DTT, 5 μM Trichostatin A, 1 mM sodium orthovanadate, 1 mM PMSF, 1 mM NaF and complete protease inhibitors (Roche Applied Science)]. Cellular lysates were prepared and the protein concentration was determined using the Pierce protein assay kit. For immunoblotting, an aliquot of total lysate (50 μg of proteins) in 2× SDS-PAGE sample buffer (1:1 v/v) was electrophoresed and transferred to a nitrocellulose filter. Filters were incubated with appropriate primary antibody in Tris-buffered saline (TBS, pH 7.5) and 5% skim milk or 5% BSA overnight. After washing, the filter was incubated with secondary antibody in TBS-Tween-20 (TBS-T) buffer for 1 h. Protein bands were visualized by an ECL detection system.

For Western blot analysis of fission yeast proteins, cells were washed once with water prior to adding cold stop buffer (150 mM NaCl, 50 mM NaF, 10 mM EDTA, 1 mM NaN_3_, pH 8.0). Cells were then collected by centrifugation and resuspended in 3 volumes of HB buffer (25 mM MOPS, pH7.2, 60 mM β-glycerophosphate, 15 mM p-nitrophenyl phosphate, 15 mM MgCI_2_, 15 mM EGTA, 1% Triton X-100, 1 mM DTT). A mixture of protease inhibitors (1 mM PMSF, 20 μg/ml leupeptin, 40 μg/ml aprotinin and 0.1 mM sodium vanadate) and a commercial complete mini protease inhibitor cocktail (Roche, one tablet per 7 ml) was added immediately before lysis by glass-bead agitation. The cells were disrupted for 60 sec using a bead beater (Biospec Products, Bartlesville, OK). Cell breakage was checked under the microscope and disruption was repeated 3–5 times if necessary. Protein concentration was measured using the BCA protein assay kit (Pierce). Equal amounts of protein (30 μg) were resolved on a SDS-polyacrylamide gel and transferred to a nitrocellulose membrane. HIV-1 Vpr or Hsp16 protein levels were assayed by immunoblotting procedures using anti-Vpr serum (generated in our laboratory) and anti-Hsp16 serum (Paul Young's laboratory), respectively. Immunoblots were developed using the enhanced chemiluminescent (ECL) system (Pierce).

## List of abbreviations

HIV-human immunodeficiency virus

Vpr-viral protein R

Hsp-heat shock protein

Hsf-heat shock factor

*S. pombe*-*Schizosaccharomyces pombe*

MDMs-monocyte-derived macrophages

PMB-polymyxin B

GFP-green fluorescent protein

## Competing interests

The author(s) declare that they have no competing interests.

## Authors' contributions

ZB did the initial analysis of Hsp16 and carried out the experiments to test the effect of Vpr on Hsp16 downregulation. DL carried out the Hsf experiments and viral infections in T-lymphocytes. JH isolated Hsp16 as the multicopy suppressor of Vpr. EA carried out the macrophage experiments. LT performed Western blot analysis on the effect of Vpr on Hsp16 at constant high temperature. PGY assisted in designing the yeast experiments and proof-read the manuscript. MB assisted in designing the mammalian experiments and drafted the manuscript. RYZ designed, directed and wrote the manuscript.

## References

[B1] Tristem M, Purvis A, Quicke DL (1998). Complex evolutionary history of primate lentiviral vpr genes. Virol.

[B2] Tristem M, Marshall C, Karpas A, Hill F (1992). Evolution of the primate lentiviruses: evidence from vpx and vpr. EMBO J.

[B3] Le Rouzic E, Benichou S (2005). The Vpr protein from HIV-1: distinct roles along the viral life cycle. Retrovirology.

[B4] Altfeld M, Addo MM, Eldridge RL, Yu XG, Thomas S, Khatri A, Strick D, Phillips MN, Cohen GB, Islam SA (2001). Vpr is preferentially targeted by CTL during HIV-1 infection. J Immunol.

[B5] Mothe BR, Horton H, Carter DK, Allen TM, Liebl ME, Skinner P, Vogel TU, Fuenger S, Vielhuber K, Rehrauer W (2002). Dominance of CD8 responses specific for epitopes bound by a single major histocompatibility complex class I molecule during the acute phase of viral infection. J Virol.

[B6] Di Marzio P, Choe S, Ebright M, Knoblauch R, Landau NR (1995). Mutational analysis of cell cycle arrest, nuclear localization and virion packaging of human immunodeficiency virus type 1 Vpr. J Virol.

[B7] Heinzinger N, Bukinsky M, Haggerty S, Ragland A, Kewalramani V, Lee M, Gendelman H, Ratner L, Stevenson M, Emerman M (1994). The Vpr protein of human immunodeficiency virus type 1 influences nuclear localization of viral nucleic acids in nondividing host cells. Proc Nat Acad Sci USA.

[B8] Goh WC, Rogel ME, Kinsey CM, Michael SF, Fultz PN, Nowak MA, Hahn BH, Emerman M (1998). HIV-1 Vpr increases viral expression by manipulation of the cell cycle: a mechanism for selection of Vpr in vivo. Nat Med.

[B9] Gibbs JS, Lackner AA, Lang SM, Simon MA, Sehgal PK, Daniel MD, Desrosiers RC (1995). Progression to AIDS in the absence of a gene for *vpr *or *vpx*. J Virol.

[B10] Lang SM, Weeger M, Stahl-Hennig C, Coulibaly C, Hunsmann G, Muller J, Muller-Hermelink H, Fuchs D, Wachter H, Daniel MM (1993). Importance of *vpr *for infection of rhesus monkeys with simian immunodeficiency virus. J Virol.

[B11] Zhao Y, Chen M, Wang B, Yang J, Elder RT, Song X-q, Yu M, Saksena N (2002). Functional conservation of HIV-1 Vpr and variability in a mother-child pair of long-term non-progressors. Virus Res.

[B12] Somasundaran M, Sharkey M, Brichacek B, Luzuriaga K, Emerman M, Sullivan JL, Stevenson M (2002). Evidence for a cytopathogenicity determinant in HIV-1 Vpr. Proc Natl Acad Sci USA.

[B13] He J, Choe S, Walker R, Di Marzio PD, Morgan DO, Landau NR (1995). Human immunodeficiency virus type 1 viral protein R (Vpr) arrests cells in the G2 phase of the cell cycle by inhibiting p34cdc2 activity. J Virol.

[B14] Jowett JB, Planelles V, Poon B, Shah NP, Chen M, Chen ISY (1995). The human immunodeficiency virus type 1 *vpr *gene arrests infected T cells in the G2 + M phase of the cell cycle. J Virol.

[B15] Li G, Elder RT, Qin K, Park HU, Liang D, Zhao RY (2007). PP2A dependent and independent pathways for ATR phosphorylation of Chk1. J Biol Chem.

[B16] Re F, Braaten D, Franke EK, Luban J (1995). Human immunodeficiency virus type 1 Vpr arrests the cell cycle in G2 by inhibiting the activation of p34^cdc2^-cyclin B. J Virol.

[B17] Yao XJ, Rougeau N, Duisit G, Lemay J, Cohen EA (2004). Analysis of HIV-1 Vpr determinants responsible for cell growth arrest in Saccharomyces cerevisiae. Retrovirology.

[B18] Poon B, Jowett JB, Stewart SA, Armstrong RW, Rishton GM, Chen IS (1997). Human immunodeficiency virus type 1 *vpr *gene induces phenotypic effects similar to those of the DNA alkylating agent, nitrogen mustard. J Virol.

[B19] Poon B, Grovit-Ferbas K, Stewart SA, Chen ISY (1998). Cell cycle arrest by Vpr in HIV-1 virions and insensitivity to antiretroviral agents. Science.

[B20] Chen M, Elder RT, Yu M, O'Gorman MG, Selig L, Benarous R, Yamamoto A, Zhao Y (1999). Mutational analysis of Vpr-induced G2 arrest, nuclear localization, and cell death in fission yeast. J Virol.

[B21] Elder RT, Yu M, Chen M, Edelson S, Zhao Y (2000). Cell cycle G2 arrest induced by HIV-1 Vpr in fission yeast (*Schizosaccharomyces pombe*) is independent of cell death and early genes in the DNA damage checkpoint. Virus Res.

[B22] Nishizawa M, Kamata M, Katsumata R, Aida Y (2000). A carboxy-terminally truncated form of the human immunodeficiency virus type 1 vpr protein induces apoptosis via G(1) cell cycle arrest. J Virol.

[B23] Waldhuber MG, Bateson M, Tan J, Greenway AL, McPhee DA (2003). Studies with GFP-Vpr fusion proteins: induction of apoptosis but ablation of cell-cycle arrest despite nuclear membrane or nuclear localization. Virology.

[B24] Lum JJ, Cohen OJ, Nie Z, Weaver JG, Gomez TS, Yao XJ, Lynch D, Pilon AA, Hawley N, Kim JE (2003). Vpr R77Q is associated with long-term nonprogressive HIV infection and impaired induction of apoptosis. J Clin Invest.

[B25] Andersen JL, Zimmerman ES, DeHart JL, Murala S, Ardon O, Blackett J, Chen J, Planelles V (2005). ATR and GADD45alpha mediate HIV-1 Vpr-induced apoptosis. Cell Death Differ.

[B26] Andersen JL, Dehart JL, Zimmerman ES, Ardon O, Kim B, Jacquot G, Benichou S, Planelles V (2006). HIV-1 Vpr-Induced Apoptosis Is Cell Cycle Dependent and Requires Bax but Not ANT. PLoS Pathog.

[B27] Benko Z, Liang D, Agbottah E, Hou J, Chiu K, Yu M, Innis S, Reed P, Kabat W, Elder RT (2004). Anti-Vpr activity of a yeast chaperone protein. J Virol.

[B28] Gu J, Emerman M, Sandmeyer S (1997). Small heat shock protein suppression of Vpr-induced cytoskeletal defects in budding yeast. Mol Cell Biol.

[B29] Vodicka MA, Koepp DM, Silver PA, Emerman M (1998). HIV-1 Vpr interacts with the nuclear transport pathway to promote macrophage infection. Genes Dev.

[B30] Zhao Y, Lieberman HB (1995). *Schizosaccharomyces pombe*: a model for molecular studies of eukaryotic genes. DNA Cell Biol.

[B31] Zhao Y, Elder RT (2000). Yeast perspectives on HIV-1 Vpr. Front Biosci.

[B32] Elder RT, Benko Z, Zhao Y (2002). HIV-1 Vpr modulates cell cycle G2/M transition through an alternative cellular mechanism other than the classic mitotic checkpoints. Front Biosci.

[B33] Zhao RY, Bukrinsky M, Elder RT (2005). HIV-1 viral protein R (Vpr) & host cellular responses. Indian J Med Res.

[B34] Bukrinsky M, Zhao Y (2004). Heat-shock proteins reverse the G2 arrest caused by HIV-1 viral protein R. DNA Cell Biol.

[B35] Taricani L, Feilotter HE, Weaver C, Young PG (2001). Expression of *hsp16 *in response to nucleotide depletion is regulated via the *spc1 *MAPK pathway in *Schizosaccharomyces pombe*. Nucleic Acids Res.

[B36] Maundrell K (1993). Thiamine-repressible expression vectors pREP and pRIP for fission yeast. Gene.

[B37] Zhao Y, Cao J, O'Gorman MRG, Yu M, Yogev R (1996). Effect of human immunodeficiency virus Type 1 protein R (*vpr*) gene expression on basic cellular functions of fission yeast *Schizosaccharomyces pombe*. J Virol.

[B38] Gummuluru S, Emerman M (1999). Cell cycle- and Vpr-mediated regulation of human immunodeficiency virus type 1 expression in primary and transformed T-cell lines. J Virol.

[B39] Yao XJ, Mouland AJ, Subbramanian RA, Forget J, Rougeau N, Bergeron D, Cohen EA (1998). Vpr stimulates viral expression and induces cell killing in human immunodeficiency virus type 1-infected dividing Jurkat T cells. J Virol.

[B40] Vanitharani R, Mahalingam S, Rafaeli Y, Singh SP, Srinivasan A, Weiner DB, Ayyavoo V (2001). HIV-1 Vpr transactivates LTR-directed expression through sequences present within -278 to -176 and increases virus replication in vitro. Virology.

[B41] Popovic M, Sarngadharan MG, Read E, Gallo RC (1984). Detection, isolation, and continuous production of cytopathic retroviruses (HTLV-III) from patients with AIDS and pre-AIDS. Science.

[B42] Bourbigot S, Beltz H, Denis J, Morellet N, Roques BP, Mely Y, Bouaziz S (2005). The C-terminal domain of the HIV-1 regulatory protein Vpr adopts an antiparallel dimeric structure in solution via its leucine-zipper-like domain. Biochem J.

[B43] Roumier T, Vieira HL, Castedo M, Ferri KF, Boya P, Andreau K, Druillennec S, Joza N, Penninger JM, Roques B, Kroemer G (2002). The C-terminal moiety of HIV-1 Vpr induces cell death via a caspase-independent mitochondrial pathway. Cell Death Differ.

[B44] Nara PL, Fischinger PJ (1988). Quantitative infectivity assay for HIV-1 and -2. Nature.

[B45] Gao B, Tsan MF (2003). Endotoxin contamination in recombinant human heat shock protein 70 (Hsp70) preparation is responsible for the induction of tumor necrosis factor alpha release by murine macrophages. J Biol Chem.

[B46] Gallo GJ, Prentice H, Kingston RE (1993). Heat shock factor is required for growth at normal temperatures in the fission yeast Schizosaccharomyces pombe. Mol Cell Biol.

[B47] Elder RT, Yu M, Chen M, Zhu X, Yanagida M, Zhao Y (2001). HIV-1 Vpr induces cell cycle G2 arrest in fission yeast (*Schizosaccharomyces pombe*) through a pathway involving regulatory and catalytic subunits of PP2A and acting on both Wee1 and Cdc25. Virology.

[B48] Albano CR, Randers-Eichhorn L, Bentley WE, Rao G (1998). Green fluorescent protein as a real time quantitative reporter of heterologous protein production. Biotechnol Prog.

[B49] Li X, Zhao X, Fang Y, Jiang X, Duong T, Fan C, Huang CC, Kain SR (1998). Generation of destabilized green fluorescent protein as a transcription reporter. J Biol Chem.

[B50] Gallo GJ, Schuetz TJ, Kingston RE (1991). Regulation of heat shock factor in Schizosaccharomyces pombe more closely resembles regulation in mammals than in Saccharomyces cerevisiae. Mol Cell Biol.

[B51] Wang L, Mukherjee S, Jia F, Narayan O, Zhao LJ (1995). Interaction of virion protein Vpr of human immunodeficiency virus type 1 with cellular transcription factor Sp1 and trans-activation of viral long terminal repeat. J Biol Chem.

[B52] Zhao Y, Yu M, Chen M, Elder RT (1998). Cell cycle G2 arrest and cell death are independent functions of HIV-1 Vpr. Proceedings of 12th World AIDS Conference, Geneva, Switzerland.

[B53] Jacotot E, Ravagnan L, Loeffler M, Ferri KF, Vieira HL, Zamzami N, Costantini P, Druillennec S, Hoebeke J, Briand JP (2000). The HIV-1 viral protein R induces apoptosis via a direct effect on the mitochondrial permeability transition pore. J Exp Med.

[B54] Rajan D, Wildum S, Rucker E, Schindler M, Kirchhoff F (2006). Effect of R77Q, R77A and R80A changes in Vpr on HIV-1 replication and CD4 T cell depletion in human lymphoid tissue ex vivo. Aids.

[B55] Connor RI, Chen BK, Choe S, Landau NR (1995). Vpr is required for efficient replication of human immunodeficiency virus type-1 in mononuclear phagocytes. Virol.

[B56] Sherman MP, de Noronha CM, Eckstein LA, Hataye J, Mundt P, Williams SA, Neidleman JA, Goldsmith MA, Greene WC (2003). Nuclear export of Vpr is required for efficient replication of human immunodeficiency virus type 1 in tissue macrophages. J Virol.

[B57] Levy DN, Refaeli Y, MacGregor RR, Weiner DB (1994). Serum Vpr regulates productive infection and latency of human immunodeficiency virus type 1. Proc Nat Acad Sci USA.

[B58] Stewart SA, Poon B, Jowett JB, Chen IS (1997). Human immunodeficiency virus type 1 Vpr induces apoptosis following cell cycle arrest. J Virol.

[B59] Humphrey T, Enoch T (1998). Sum1, a highly conserved WD-repeat protein, suppresses S-M checkpoint mutants and inhibits the osmotic stress cell cycle response in fission yeast. Genetics.

[B60] Zhao Y, Yu M, Chen M, Elder RT, Yamamoto A, Cao J (1998). Pleiotropic effects of HIV-1 protein R (Vpr) on morphogenesis and cell survival in fission yeast and antagonism by pentoxifylline. Virology.

[B61] Masuda M, Nagai Y, Oshima N, Tanaka K, Murakami H, Igarashi H, Okayama H (2000). Genetic studies with the fission yeast Schizosaccharomyces pombe suggest involvement of *wee1*, *ppa2*, and *rad24 *in induction of cell cycle arrest by human immunodeficiency virus type 1 Vpr. J Virol.

[B62] Lee M, Nurse P (1988). Cell cycle control genes in fission yeast and mammalian cells. Trends Genet.

[B63] Nurse P, Thuriaux P, Nasmyth K (1976). Genetic control of the cell division cycle in the fission yeast Schizosaccharomyces pombe. Mol Gen Genet.

[B64] Zhao Y, Elder RT, Chen M, Cao J (1998). Fission yeast expression vectors adapted for large scale cloning and GFP fusion with positive screening. BioTechniques.

[B65] Schmidtmayerova H, Nuovo GJ, Bukrinsky M (1997). Cell proliferation is not required for productive HIV-1 infection of macrophages. Virology.

[B66] Gendelman HE, Orenstein JM, Martin MA, Ferrua C, Mitra R, Phipps T, Wahl LA, Lane HC, Fauci AS, Burke DS (1988). Efficient isolation and propagation of human immunodeficiency virus on recombinant colony-stimulating factor 1-treated monocytes. J Exp Med.

